# ACPA fine-specificity profiles in early rheumatoid arthritis patients do not correlate with clinical features at baseline or with disease progression

**DOI:** 10.1186/ar4322

**Published:** 2013-10-01

**Authors:** Joyce JBC van Beers, Annemiek Willemze, Jeroen J Jansen, Gerard HM Engbers, Martin Salden, Jos Raats, Jan W Drijfhout, Annette HM van der Helm-van Mil, Rene EM Toes, Ger JM Pruijn

**Affiliations:** 1 Department of Biomolecular Chemistry, Institute for Molecules and Materials and Nijmegen Center for Molecular Life Sciences, Radboud University Nijmegen, PO Box 9101, NL-6500 HB Nijmegen, The Netherlands; 2 Department of Rheumatology, Leiden University Medical Center, PO Box 9600, NL-2300 RC Leiden, The Netherlands; 3 Department of Analytical Chemistry, Institute for Molecules and Materials, Radboud University Nijmegen, PO Box 9010, NL-6500 GL Nijmegen, The Netherlands; 4 Ssens BV, Pantheon 1, NL-7521 PR, Enschede, The Netherlands; 5 Euro Diagnostica AB, Toernooiveld 1, NL-6525 ED Nijmegen, The Netherlands; 6 ModiQuest BV, Pivot Park RE2142, Molenweg 79, NL-5349 AC Oss, The Netherlands; 7 Department of Immunohematology and Blood Transfusion, Leiden University Medical Center, PO Box 9600, NL-2300 RC Leiden, The Netherlands

## Abstract

**Introduction:**

Autoantibodies against citrullinated peptides/proteins (ACPA) are found in approximately 75% of the sera of patients with rheumatoid arthritis (RA). The RA-specific ACPA are frequently present prior to disease onset and their presence associates with a more erosive disease course. ACPA can therefore be used to aid the diagnosis and prognosis of RA. Recently, it became clear that ACPA are very heterogeneous, both in an individual patient and among different patients. The aim of this study was to investigate whether clinically meaningful ACPA profiles exist in early RA patients.

**Methods:**

Twenty citrullinated peptides and the corresponding non-citrullinated control peptides were immobilized on microarray sensor chips. Sera from 374 early arthritis patients were analyzed by surface plasmon resonance imaging (*i*SPR) of biomolecular interactions on the sensor chip.

**Results:**

Cluster analysis of the reactivities with the citrullinated peptides, after subtraction of the reactivities with the corresponding control peptides confirmed the heterogeneity of the ACPA response in RA and revealed 12 distinct ACPA profiles. The association of the 5 most frequent profiles with clinical features at diagnosis and during the disease course was examined, showing no statistically significant associations.

**Conclusions:**

Compared to the detection of ACPA in RA sera by CCP-based assays, ACPA profiling in early arthritis patients did not reveal associations with disease activity and progression scores.

## Introduction

Autoantibodies against citrullinated proteins (ACPAs) are specifically found in approximately 75% of rheumatoid arthritis (RA) patients [1]. Citrullination is the posttranslational conversion of peptidylarginine into peptidylcitrulline, which is catalyzed by peptidylarginine deiminase in a calcium-dependent manner [2]. Accumulating evidence suggests that citrullinated proteins and ACPAs are directly involved in the pathophysiology of RA. Several citrullinated antigens (for example, fibrinogen, α-enolase, vimentin, collagen type II and fibronectin) have been identified in the inflamed joints of RA patients [3-9]. Citrullinated autoepitopes of these proteins provide the opportunity to investigate the ACPA response to genuine autoantigens and to assess the production of autoantibodies to these epitopes early in disease.

The diagnosis of RA relies in part on the detection of ACPA, for example, by the most commonly applied cyclic citrullinated peptide 2 (CCP2) test [10]. It has been demonstrated that ACPAs are already present prior to disease onset and that they are associated with a more severe course of RA [11-13]. Although just about all ACPAs are reactive in the CCP2 test, diverse reactivity is observed when other citrullinated molecules are used, for example, peptides derived from fibrinogen and vimentin [14]. This indicates that the ACPA response in RA is heterogeneous, with diverse patterns of reactivity to distinct citrullinated epitopes. Currently, RA patients are classified into two distinct groups: anti-CCP2-positive and anti-CCP2-negative. It remains to be investigated whether the ACPA fine specificity may improve diagnosis and/or prognosis [15,16]. Because the ACPA response is very heterogeneous and differs among individual RA patients, identifying ACPA profiles and analyzing their association with clinical features, rather than looking at the ACPA-positive RA patients as one group, may facilitate the subclassification of patients and may aid the development of “patient-tailored” therapies in the future. It is interesting to note that low and intermediate pretreatment levels of ACPA appear to be associated with a more favorable response to methotrexate treatment in recent onset anti-CCP-positive arthritis, whereas high levels are associated with an insufficient response [17].

ACPA profiles can be determined most efficiently by using multiplex assays, in which multiple citrullinated antigens are tested simultaneously [18-21]. An example of such a multiplex assay is microarray surface plasmon resonance imaging (*i*SPR), which is a label-free method used to detect biomolecular interactions in real time. The fully automated *i*SPR analysis requires only a minimal amount of serum, and the microarray-containing sensor chips can be regenerated and used repeatedly. The use of *i*SPR for monitoring autoantibody binding to different citrullinated targets was first described by Lokate and coworkers [22], and later citrullinated B-cell epitopes in fibrinogen were successfully mapped with the use of this technology [23]. A recently introduced continuous flow spotting system to generate the *i*SPR microarrays not only improved the robustness and reproducibility of the arrays but also allowed much higher spotting volumes and an increased number of spots per array.

In our present study, we applied an *i*SPR microarray to identify ACPA profiles in early arthritis patients using a microarray containing 20 citrullinated peptides, as well as their noncitrullinated counterparts, and compared these profiles with clinical features of the patients.

## Methods

### Patient information

Early arthritis sera (*n* = 374) were collected at the Department of Rheumatology of the Leiden University Medical Center (Leiden, The Netherlands) from the Leiden Early Arthritis Clinic. All patients fulfilled the American Rheumatism Association 1987 revised criteria for the classification of RA within one year of follow-up [24]. The Leiden Early Arthritis Clinic is an inception cohort of patients with recent-onset arthritis (symptom duration less than two years) that was started at the Department of Rheumatology of the Leiden University Medical Center in 1993 and was described in detail previously [25].

At the time of inclusion, patients were asked about their joint symptoms and underwent a physical examination. At baseline and at the yearly follow-up visits, blood samples were taken for routine diagnostic laboratory screening and serum was stored at −70°C. Written informed consent was obtained from all participants. The study was approved by the local medical ethics committee of the Leiden University Medical Center.

Sera from healthy individuals (*n* = 10) were collected at the Sanquin Blood Bank in Nijmegen, The Netherlands. Sera were stored at −70°C until use. Total immunoglobulin G anti-CCP2 antibodies in RA sera were measured using the Immunoscan CCPlus enzyme-linked immunosorbent assay (ELISA) kit (Euro Diagnostica AB, Malmö, Sweden). Samples with a value greater than 25 U/ml were considered positive.

### Preparation of the microarrays and surface plasmon resonance imaging analyses

Peptides (Table 1) were synthesized by performing a solid-phase procedure using 9-fluorenylmethyloxycarbonyl chemistry as described previously [26]. The peptides were at least 90% pure, as deduced from their elution pattern on reverse-phase high-performance liquid chromatography. Biotinylated peptides were immobilized on an *i*SPR chip containing a streptavidin hydrogel linked to a gold layer on a glass surface (Ssens BV, Enschede, The Netherlands) (Additional file 1: Figure S1A). In short, immobilization of the peptides was initiated by spotting the peptides on the surface of the sensor discs using a continuous flow microspotter (Watsatch Microfluidics, Salt Lake City, UT, USA) for one hour to generate a 48-spot microarray (Additional file 1: Figure S1B) containing 48 spots of approximately 0.5 mm^2^ each (Additional file 1: Figure S1C).

**Table 1:** **Peptide sets immobilized on the microarrays for surface plasmon resonance imaging analysis**^**a**^

**Set**		**Origin**	**Peptide sequence**	**Position of citrulline residues**^**b**^
1	A	α-enolase (cyclic)	CKIHA**R**EIFDS**R**GNPTVECZO	
	B		CKIHA**X**EIFDS**X**GNPTVECZO	9, 15
2	A	β-actin	MKILTE**R**GYSFTTAE**R**EIVRDIKEKLZO	
	B		MKILTE**X**GYSFTTAE**X**EIVXDIKEKLZO	196, 206, 210
3	A	CCP2-like (cyclic)	^c^	
	B			n.r.
4	A	Collagen type II	GLPGVKGH**R**GYPGLDGAZO	
	B		GLPGVKGH**X**GYPGLDGAZO	290
5	A	Fibrinogen	FLAEGGGV**R**GPRVVERHZO	
	B		FLAEGGGV**X**GPRVVERHZO	α35^d^
6	A	Fibrinogen	TSSTSYN**R**GDSTFZO	
	B		TSSTSYN**X**GDSTFZO	α591^d^
7	A	Fibrinogen	NEEGFFSA**R**GHRPLDKKZO	
	B		NEEGFFSA**X**GHRPLDKKZO	β44^e^
8	A	Fibrinogen	EEAPSL**R**PAPPPISGGGY**R**A**R**PAKAAAZO	
	B		EEAPSL**X**PAPPPISGGGY**X**A**X**PAKAAAZO	β60, 72, 74^e^
9	A	Fibronectin	LTVGLT**RR**GQPRQYZO	
	B		LTVGLT**XX**GQPRQYZO	1,035, 1,036
10	A	Fibronectin	YNQYSQ**R**YHQRTNZO	
	B		YNQYSQ**X**YHQRTNZO	2356
11	A	Filaggrin (cfc1; cyclic)	HQCEST**R**GRSRGRCGRSGSZO	^f^
	B		HQCEST**X**GRSRGRCGRSGSZO	
12	A	MNDA	KLTSEA**R**G**R**IPVAQKZO	
	B		KLTSEA**X**G**X**IPVAQKZO	127, 129
13	A	Peptide library	^c^	
	B			n.r.
14	A	Peptide library	^c^	
	B			n.r.
15	A	Peptide library	^c^	
	B			n.r.
16	A	Peptide library	^c^	
	B			n.r.
17	A	Vimentin	MST**R**SVSSSSY**RR**MFGGPZO	
	B		MST**X**SVSSSSY**XX**MFGGPZO	4, 12, 13
18	A	Vimentin	GVYAT**R**SSAV**R**L**R**SSVPGZO	
	B		GVYAT**X**SSAV**X**L**X**SSVPGZO	64, 69, 71
19	A	Vimentin	LTAAL**R**DV**R**QQYESZO	
	B		LTAAL**X**DV**X**QQYESZO	270, 273
20	A	Vimentin	SLNL**R**ETNLDSLPLVDZO	
	B		SLNL**X**ETNLDSLPLVDZO	424

^a^CCP2, cyclic citrullinated peptide 2; MNDA, myeloid cell nuclear differentiation antigen; n.r., not relevant; **O**, biotinylated lysine; **X**, citrulline; **Z**, aminohexanoic acid. ^b^Amino acid position in the corresponding polypeptide relative to the N-terminal methionine of the primary translation product. ^c^Sequence is defined in patent EP2071335. ^d^α-Chain of fibrinogen. ^e^β-Chain of fibrinogen. ^f^Derived from sequence presented in [27].

Prior to use, sera were diluted 50-fold in phosphate-buffered saline (PBS) with 0.075% Tween 80. Incubation, washing and regeneration were performed in an automated way using liquid-handling procedures in a comprehensive surface plasmon resonance imaging system for multiplexing 96 biomolecular interactions (IBIS-MX96, IBIS Technologies, Enschede, The Netherlands). Using the IBIS-MX96 system, a diluted serum sample plug of 80 μl was injected and 20 μl were guided backward and forward over the microarray in a flow cell at a speed of 30 μl/s to allow autoantibody binding to the immobilized peptides for 40 minutes. The serum sample plug was flanked by two air plugs to prevent the diffusion of serum components into the buffer. Between the association phase and the regeneration phase, the flow cell was rinsed with PBS with 0.075% Tween 80 for eight minutes. The array was regenerated twice by two consecutive injections of 80 μl of 10 mM glycine∙HCl, pH 2.5, for 30 seconds.

A mixture of two monoclonal ACPAs (10 μg/ml of each antibody; Modiquest BV, Oss, The Netherlands) and serum from an anti-CCP2-positive RA serum (50-fold dilution; Radboud University Medical Centre Nijmegen) as a control to verify successful immobilization and as a standard for different microarrays. To obtain quantitative results for antibody binding to the peptides, the data were analyzed using SPRint software (IBIS Technologies, Enschede, The Netherlands).

### Data analysis

Hierarchical clustering software Cluster 3.0 was used to identify ACPA profiles, and the results were displayed using Java TreeView software (Stanford University, Stanford, CA, USA).

The association of the different clusters of patients with categorical baseline characteristics (for example, gender, CCP2) was studied using Pearson’s χ^2^ test. The independent samples Kruskal-Wallis test was performed to address associations with clinical phenotype (Disease Activity Score, Visual Analogue Scale (VAS) score, swollen joint counts, tender joint count (Ritchie index), erythrocyte sedimentation rate (ESR) and C-reactive protein (CRP)).

Furthermore, the association of the different clusters and joint damage over time was studied. Radiographs of hands and feet, taken at baseline and yearly thereafter during seven years of follow-up, were assessed according to the Sharp/van der Heijde score (SHS) with known time order by an experienced reader who was blinded to any clinical information. The within-reader intraclass correlation coefficient was 0.91. This method takes advantage of the longitudinal, repetitive character of the data and does not exclude patients with incomplete follow-up data, thus avoiding selection bias.

In a multivariate normal regression model with radiological score used as the response variable, the effect of time was entered as a factor to fit the nonlinear slope of joint destruction. The components were entered with an interaction term with time used as a continuous variable to test the effect of the components over time. Age, gender and inclusion period (a proxy for treatment strategy) were entered into the model to correct for possible confounding effects. Analyses were performed using SPSS version 17.0 software (SPSS, Inc, Chicago, IL, USA), and *P* values below 0.05 were considered statistically significant.

## Results

### Autoantibody detection in patient sera by microarray surface plasmon resonance imaging

**Figure 1:**
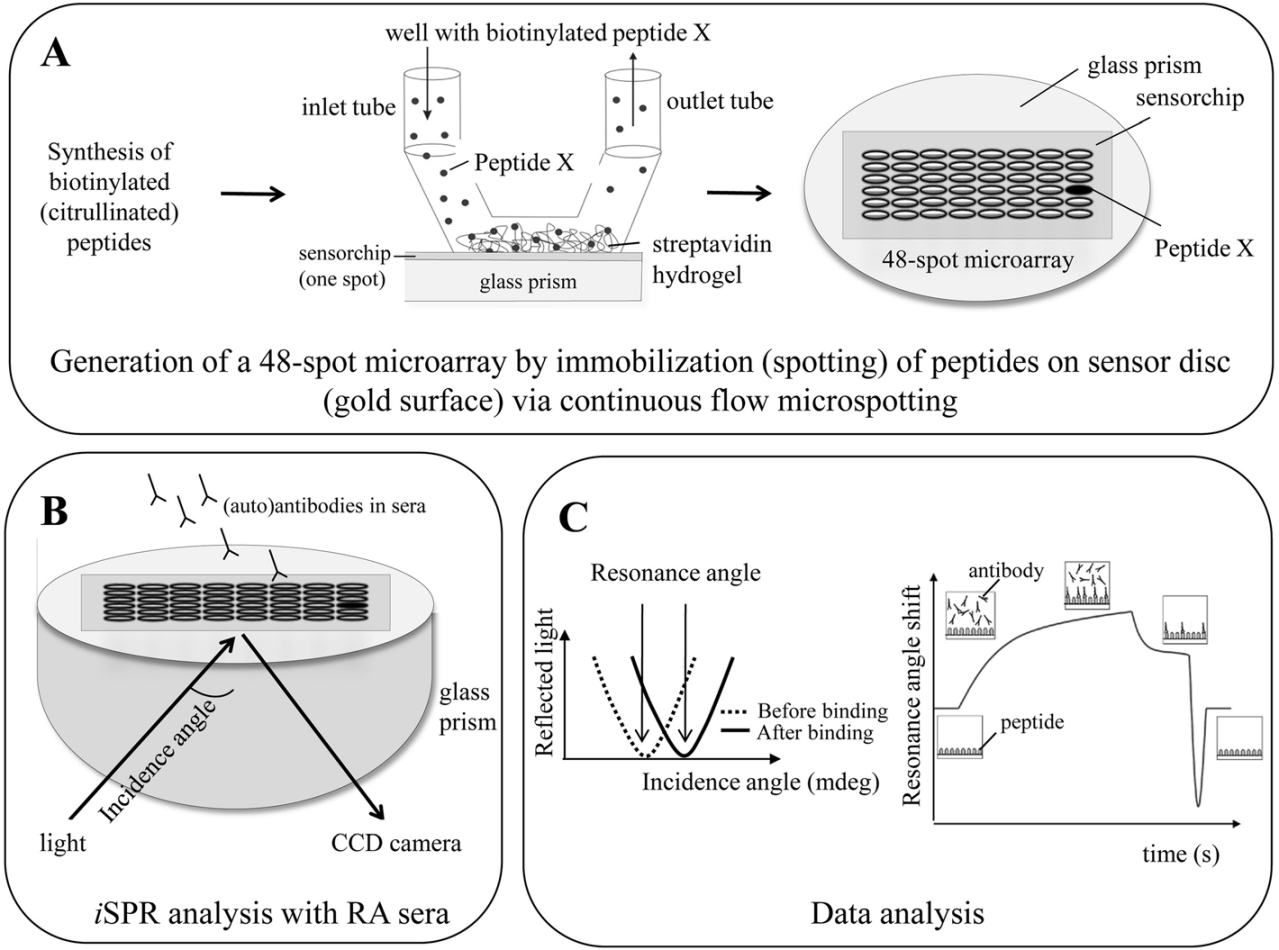
**Schematic overview of autoantibody detection by microarray surface plasmon resonance imaging. (A)** Biotinylated peptides are immobilized on a sensor chip which contains a gold surface with a streptavidin hydrogel on top. Each peptide (48 in total) is pumped back and forth along a specific position of the chip for one hour. This leads to a 48-spot microarray, in which each spot contains a different peptide. **(B)** Interactions of serum antibodies with peptides on the 48-spot microarray are detected by surface plasmon resonance imaging (*i*SPR). The incident light is reflected at the sensor chip surface and detected by a charge-coupled device (CCD) camera. **(C)** At a certain incidence angle, the amount of reflected light will be at a minimum. The binding of an antibody leads to a mass change at the surface and results in a shift in the resonance angle. The resonance angle is plotted against time to generate a sensorgram.

An overview of the method used in the present study is shown in Figure 1. The use of *i*SPR to study antibody-antigen binding has been described previously [22,23]. Since then, several adjustments have been made to the system, the most important of which is the immobilization of peptides on the microarrays. To avoid flowing out of the small droplets spotted on the surface, ligands (for example, peptides) are now placed on a sensor chip by continuous flow microspotting, in which a 48-channel printhead makes direct contact with the surface of the sensor chip. Each peptide solution is pumped back and forth along a defined spotting area of the chip for one hour. Furthermore, streptavidin-coated hydrogel sensor chips were used to immobilize C-terminal biotinylated peptides, which not only results in a similar mode of presentation of all peptides but also is less laborious. Other adjustments concern software updates improving data analysis.

Because the streptavidin-coated sensor chips were not used before for autoantibody detection by microarray *i*SPR and the streptavidin-biotin interaction is not covalent, it was necessary to determine the effects of acid-induced microarray regeneration. A standard microarray sensor chip was consecutively incubated 30 times with different analytes, with a regeneration step between each of the serum incubations. The results showed that the microarrays could be reused more than 30 times without a major reduction in resonance angle shift. In addition, the reproducibility of the microarray analyses was demonstrated by the inclusion of patient serum reactive with multiple citrullinated peptides and a mixture of monoclonal antibodies to citrullinated proteins (Additional file 1: Figure S2).

### Peptide microarrays for multiplexed detection of autoantibodies against citrullinated protein

Over the past decade, several citrullinated autoantigens targeted by ACPA have been identified (fibrinogen, vimentin, α-enolase, fibronectin and collagen type II) [3-7,9]. In initial *i*SPR experiments, we have explored the suitability of 31 peptide sets (citrulline-containing and corresponding arginine- or norleucine-containing peptides) derived from several citrullinated autoantigens. This research led to the selection of 20 peptide sets (Table 1) derived from fibrinogen (four sets), fibronectin (two sets), collagen type II (one set), vimentin (four sets), α-enolase (one set), β-actin (one set), myeloid nuclear differentiation antigen (one set) and filaggrin (one set) or isolated from randomized peptide libraries (five sets) for the generation of the standard microarray. The remaining eight spots were reserved for several highly reactive citrullinated peptides, which served as quality controls in the microarrays. On the basis of the results obtained with sera from healthy controls, a cutoff value of 10 mdeg was chosen.

### Detection of autoantibodies against citrullinated protein detection by microarray surface plasmon resonance imaging

**Figure 2:**
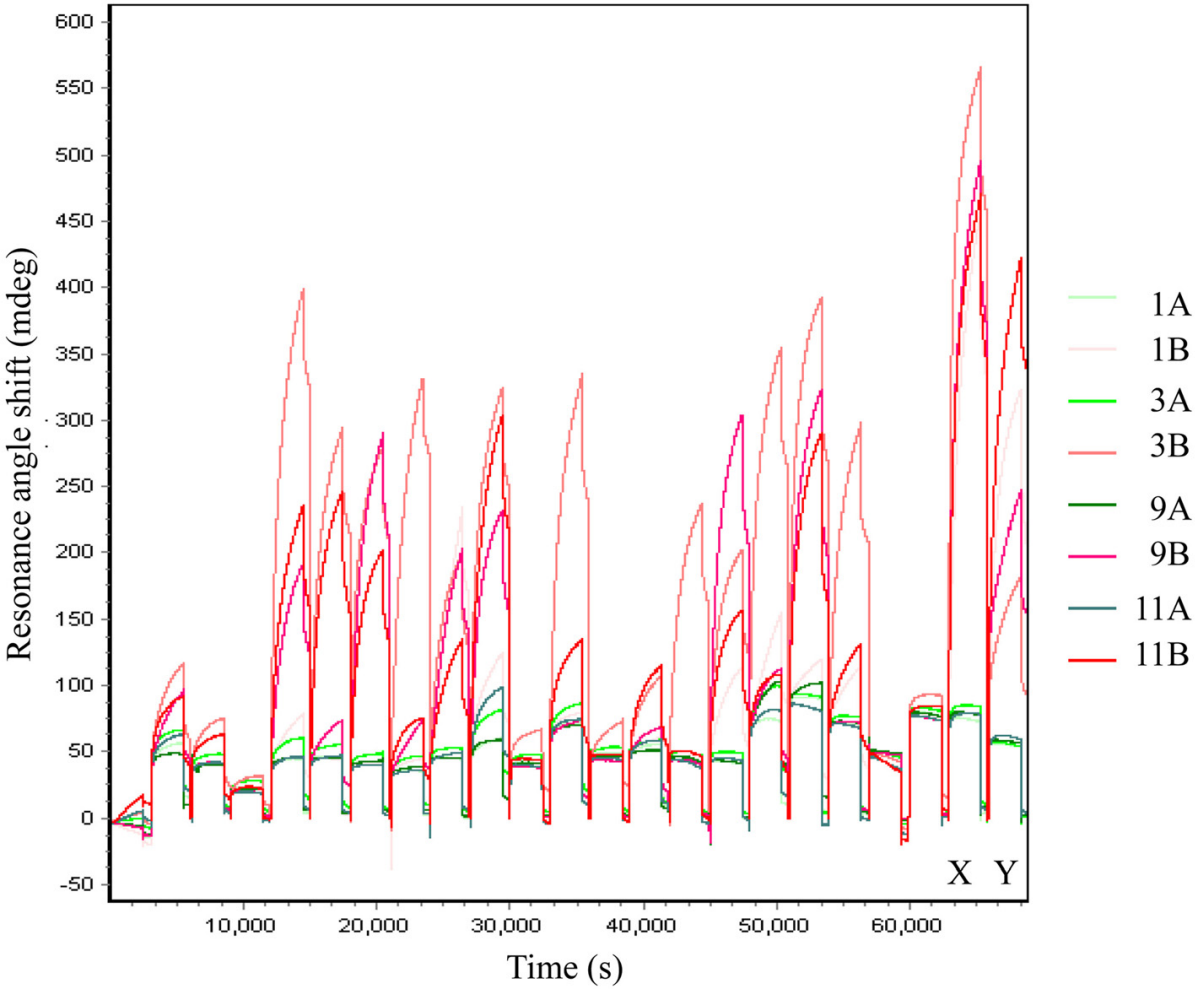
**Detection of autoantibodies against citrullinated protein in anti-cyclic citrullinated peptide 2-positive early arthritis sera by microarray surface plasmon resonance imaging.** Sensorgrams for the binding of 20 anti-CCP2-positive early arthritis sera to four peptide sets are shown. The different citrullinated peptides are depicted in different shades of red, and the arginine control peptides are shown in different shades of green. The different colors the four different citrullinated peptides illustrate the data listed in Table 1. Widely reactive autoantibodies against citrullinated protein (ACPA)-positive serum from a rheumatoid arthritis patient (X) and a mixture of two monoclonal ACPAs (Y) were used as controls.

Serum samples from 374 early arthritis patients were analyzed with *i*SPR using the synthetic peptide microarrays described above. A total of 187 of these patients were anti-CCP2-positive, and 187 were anti-CCP2-negative at baseline, as revealed in anti-CCP2 ELISA tests. As expected, various reactivity patterns were observed for these sera, and anti-CCP2-positive sera generally showed more reactivity with citrullinated peptides than with the corresponding control peptides. In addition, the *i*SPR data emphasize the heterogeneity of the ACPA response in the anti-CCP2-positive patients (Figure 2).

**Figure 3:**
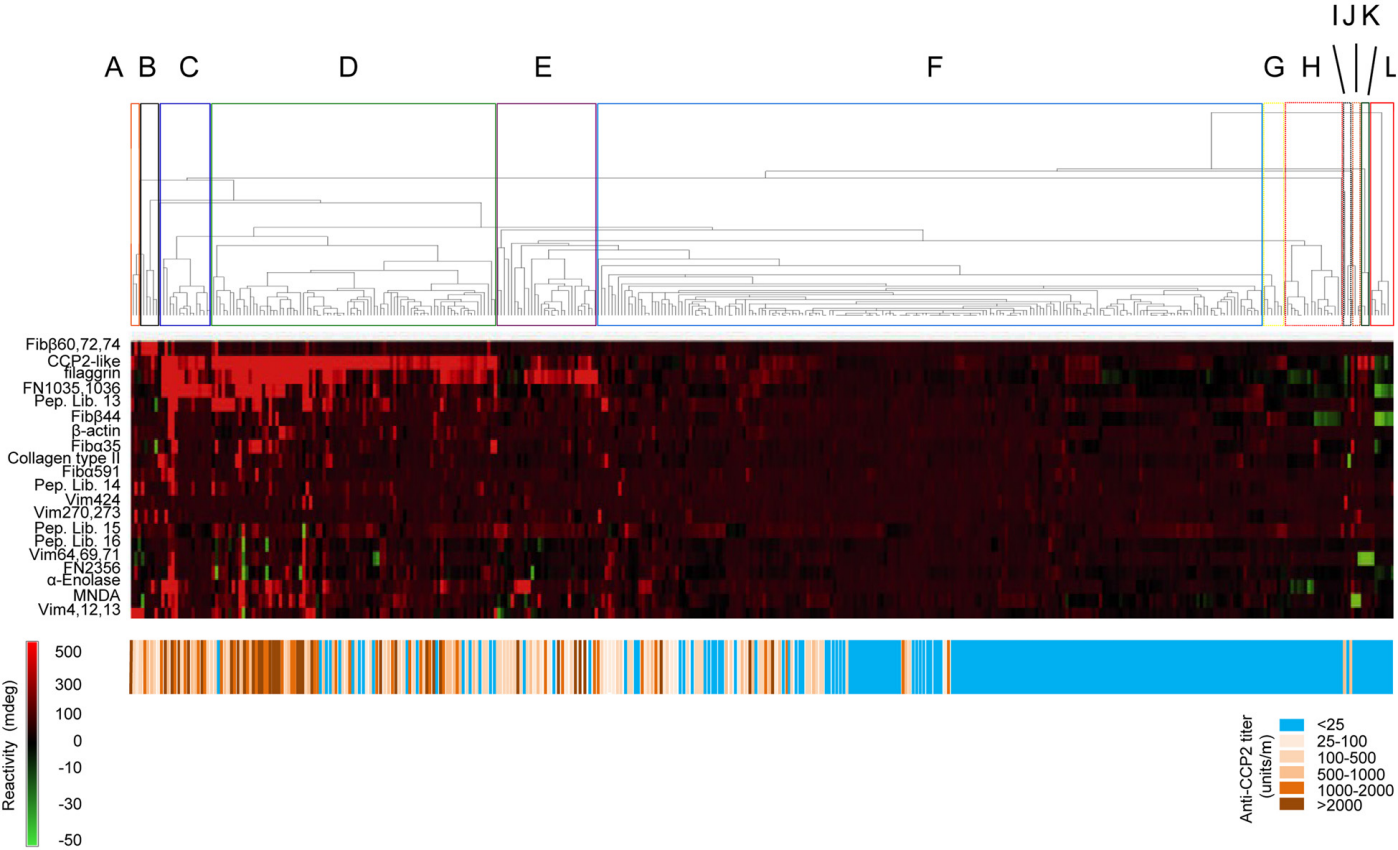
**Clustering of early arthritis patient sera based upon microarray surface plasmon resonance imaging data.** Microarray surface plasmon resonance imaging data for 374 early arthritis patient sera were subjected to cluster analysis using Cluster 3.0 software. The results were used to generate a heat map, with the results for the individual peptides given in rows (peptide numbers indicated on the right) and those for the patients given in columns. **(A)** through **(L)** On the basis of the dendrograms, 12 autoantibodies against citrullinated protein profiles were defined. Below the heat map, the anti-cyclic citrullinated peptide 2 (CCP2) reactivity is shown: anti-CCP2-positive is shown in different shades of brown and orange, and anti-CCP2-negative is shown in blue. Fib: fibrinogen; FN: fibronectin; Pep. Lib.: peptide library; MNDA: myeloid cell nuclear differentiation antigen; Vim: vimentin.

Eleven (6%) of the anti-CCP2-negative sera recognized at least one citrullinated peptide. Eight of these sera recognized one citrullinated peptide, two sera were reactive with two citrullinated peptides and one serum recognized four citrullinated peptides (Figure 3).

During the analysis, cycles of each sensor chip a mixture of two monoclonal ACPAs and a highly reactive RA serum were used as positive controls to confirm efficient peptide immobilization (Figure 2).

### Autoantibody against citrullinated protein profiling

To identify the presence of common reactivity patterns in the data set, obtained after subtracting the reactivities lobserved with the control peptides from those of the corresponding citrullinated peptides, cluster analysis was performed. On the basis of the results (Figure 3), we defined 12 different clusters of patients (A through L), some of which comprise only a limited number of patients. Remarkably, one of these clusters (F) contains both anti-CCP2-positive and anti-CCP2-negative patients. The results of principal component analysis (Additional file 1) support the differentiation in ACPA profile clusters (A through L) identified with hierarchical clustering software Cluster 3.0 (Additional file 1: Figure S3).

Five clusters (C, D, E, F and H), each consisting of more than 15 patients, were analyzed for associations with serological, genetic and clinical features. From C to H, the percentage of anti-CCP2-positive and RF-positive sera, the mean ACPA levels, the percentage of the different ACPA isotypes and the frequency of HLA-DRB1 shared epitope (SE) positivity gradually decreased (Table 2 and Additional file 1: Table S1). Similarly, the mean number of citrullinated peptides recognized decreased (Table 2). The different clusters displayed significant differences in mean ACPA levels, ACPA status and the presence of HLA-DRB1 SE alleles.

**Table 2:** **Autoantibody and disease features of patients with the five major autoantibodies against citrullinated protein profiles**^**a**^

**Group**	***n***	**Anti-CCP2+**^**b **^**(%)**	**RF+ (%)**	**Mean ACPA level (U/ml)**	**Mean number of citrullinated antigens recognized (range)**^**c,d**^	**(Ex-) smokers (%)**^**e**^	**SE+**^**f **^**(%)**^**d**^	**VAS median**^**g**^
C	15	100	93	4630	6 (1 to 16)	38	93	34
D	83	88	87	1317	3 (1 to 13)	59	80	35
E	30	87	87	861	2 (0 to 5)	66	70	34
F	198	31	45	137	0 (0 to 4)	49	53	40
H	17	0	29	14	0 (0 to 1)	60	29	50

^a^ACPA: anticitrullinated protein antibody; CCP2: cyclic citrullinated peptide 2; RF: rheumatoid factor; SE: shared epitope; VAS: Visual Analogue Scale score. ^b^Measured using the anti-CCP2 test (CCPlus Immunoscan assay). ^c^Reactivity is based upon cutoff values determined with reactivity of healthy individuals with surface plasmon resonance imaging. ^d^*P*-value < 0.01. ^**e**^*P*-value = 0.278. ^f^At least one SE allele. ^g^*P*-value = 0.958. Group differences were tested using the Kruskal-Wallis test or Pearson’s χ^2^ test.

No associations were observed between different ACPA profiles and several disease characteristics at baseline (gender, Ritchie index score, number of swollen joints, CRP level, ESR and pain sensation measured by VAS score (Table 2 and Additional file 1: Table S1). Radiological progression over seven years, studied on the basis of SHS, also was not associated with the different ACPA profiles. As expected, the cluster of patients with the highest percentage ACPA-positive patients displayed more joint damage than the ACPA-negative cluster. After stratification for ACPA status, however, no significant differences between the clusters remained.

## Discussion

In the present study, microarray *i*SPR analysis was used to identify ACPA fine-specificity profiles. Twenty peptide sets derived from eight different proteins and peptides from peptide library screenings were used to study and determine the ACPA profiles. Twelve distinct ACPA profiles were identified in three hundred seventy-four early arthritis patients. The data confirm the heterogeneity of the ACPA response in RA patients. Sera from patients with the ACPA profile with the highest percentage anti-CCP2 positivity also showed the highest ACPA levels, as well as the highest number of citrullinated peptides recognized, and were from patients with the highest percentage of SE alleles. No association with smoking was observed (Table 2).

Associations of ACPA profiles with clinical features might be helpful in determining patients’ prognosis and the development of “tailor-made” treatment, as has been demonstrated for anti-CCP2 positivity in general, which is associated with more erosive disease and for which the effectiveness of early treatment (for example, by combined drug therapy) to prevent progression of joint destruction has been demonstrated [28]. Our analyses of the five most frequently observed profiles in the early arthritis cohort did not demonstrate associations with disease activity or erosion levels.

The reactivity to multiple citrullinated antigens has been addressed in several studies, in most cases on the basis of ELISA [29-32]. In agreement with our data, in these prior studies, no association with clinical characteristics was observed [29,33]. It is still too early to conclude that ACPA profiling is clinically meaningless. The number of sera that have been analyzed is still relatively low and came from a limited number of cohorts. Larger numbers of samples, from independent cohorts and from distinct stages of disease development, need to be analyzed to validate the clustering obtained with the early arthritis sera, to generate more reliable data on correlations with clinical features and to address associations with the minor profiles.

Besides microarray *i*SPR, several alternative multiplex systems have been developed to profile biomolecular markers in RA. These include systems for multiple serum biomarkers (for example, CRP, interleukin 6) [34], multiple gene expression profiling [35] and the detection of multiple cytokines to predict disease development and the response to therapy [36-38]. In addition, several systems for autoantibody profiling have been developed, most of which are focused on multiplexed ACPA detection [19-21,38]. A comparison of the citrullinated antigens used in these multiplex assays with the molecules used in the current study is given in Additional file 1: Table S2.

One of the first multiplex ACPA profiling assays, protein/peptide microarrays on which antibody binding was detected by fluorescent secondary antibodies, was described by Hueber and coworkers [38]. The results showed that patients whose sera were predominantly reactive with citrullinated antigens carry two SE alleles and/or reported increased Health Assessment Questionnaire (HAQ) disability scores. Using our microarrays, we observed similar correlations with SE status, as noted above. Although HAQ scores (which include aspects of disability, pain, medication effects, cost of care and mortality) were not assessed in our study, the VAS scores (which register only pain sensation) were not significantly different between the patients with the five different ACPA profiles analyzed.

A more reproducible and automated variant of this system was reported by Chandra and coworkers [20]. In addition, in this system, fluorescently labeled antibodies were used to detect autoantibodies as well as cytokines bound on the microarrays. Cluster analysis of a data set obtained with this system confirmed the association of ACPA with SE-positive RA patients. Advantages of this multiplex assay are its reproducibility and the fact that RA patients can be clearly distinguished from control individuals. Disadvantages are that the number of different markers (ten) that can be measured simultaneously is limited and that the chip can be used only once.

Sokolove and colleagues developed a novel multiplex system using a bead-based assay in which 20 target antigens, including 3 arginine control proteins, 3 (*in vitro*) citrullinated proteins and 14 citrullinated peptides can be measured simultaneously [21]. They reported increased autoantibody reactivity over time as individuals approached the development of clinical RA. In agreement with previously published ELISA data [39], they observed a correlation between the increasing number of ACPA subtypes and elevation of anti-CCP2 titers. This finding is similar to our observations that patients with the ACPA profile with the highest mean ACPA levels (C) are also reactive with the highest number of citrullinated antigens (Table 2).

Recently, another microarray-based multiplex system for the detection of autoantibodies against citrullinated peptides was introduced [19]. In this system, 12 peptide sets (partially similar to the peptides used in our study; see Additional file 1: Table S3) were used as target antigens. A good correlation with results obtained by ELISA was observed. An advantage of the latter method is the ability to measure reactivities with 170 antigens simultaneously. This system was successfully used to study antibodies against citrullinated peptides in individuals prior to RA development [40]. In agreement with previously published data [39,41], the results of this study showed that the ACPA response is initially restricted but expands over time with increasing levels toward disease onset. It is important to note that, in all these systems, labeled antibodies are needed for the detection of bound antibodies.

## Conclusions

Microarray *i*SPR analysis is the only label-free, real-time detection system for autoantibodies described so far. The ability to regenerate the microarrays and to analyze more than 30 patient sera consecutively in a fully automated fashion on a single array makes this system unique among the various multiplex systems that have been developed. Moreover, only very small volumes of serum are required to measure reactivity with multiple target molecules simultaneously.

ACPA profiling using microarrays containing 20 peptide sets derived from 8 different proteins and peptides derived from peptide library screenings allowed the identification of 12 distinct ACPA profiles in 374 early arthritis patients. These data confirm the heterogeneity of the ACPA response in RA patients. ACPA profiling in these early arthritis patients did not reveal associations with disease activity and progression scores.

We conclude that microarray *i*SPR represents a suitable system for multiplexed ACPA detection in patient sera and that more analyses have to be performed to assess the predictive and/or prognostic value of ACPA profiling.

## Abbreviations

ACPA: Anticitrullinated protein antibody; CCP: Cyclic citrullinated peptide; CRP: C-reactive protein; ESR: Erythrocyte sedimentation rate; HAQ: Health assessment questionnaire; iSPR: Surface plasmon resonance imaging; LHP: PA, Palindromic arthritis; RA: Rheumatoid arthritis; RF: Rheumatoid factor; SE: Shared epitope; UA: Undifferentiated arthritis; VAS: Visual analogue scale.

## Competing interests

The authors declare that they have no competing interests.

## Authors’ contributions

JB, GE, MS, JR and GP designed the study. JB and AW performed the experiments. JB, AW, JJ, AHM, RT and GP analyzed the data. JB, AW, JJ, GE, MS, JR, JD, AHM, RT and GP interpreted the data. JD provided the synthetic peptides. All authors read and approved the final manuscript.

## Supplementary Material

Additional file 1 Supplementary Materials and Methods; Supplementary Tables S1 and S2; Supplementary Figures S1–S3. Click here for file
