# Supplementary material for: ACPA fine-specificity profiles in early rheumatoid arthritis patients do not correlate with clinical features at baseline or with disease progression
Source: Arthritis Res Ther. 2013 Oct 1;15(5):R140. doi: 10.1186/ar4322 (PMC3978944; doi:10.1186/ar4322)

SUPPLEMENTARY INFORMATION

ACPA fine-specificity profiles in early rheumatoid arthritis patients do not correlate with clinical features at baseline nor with disease progression

Joyce JBC van Beers, Annemiek Willemze, Jeroen J Jansen, Gerard HM Engbers, Martin Salden, Jos Raats, Jan W. Drijfhout, Annette HM van der Helm-van Mil, Rene EM Toes and Ger JM Pruijn

Supplementary Materials and methods

Statistical Analysis

The microarray-iSPR data was analyzed by Principal Component Analysis. This method describes all variation in the dataset in a number of variables called Principal Components (PCs), which is considerably less than the number of the peptides. As these PCs contain the relationships—*i.e.* the covariances—between the reactivity on different peptide sets, the constructed PCs will capture most of the variation among the profiles. The 'profile scores' of this model then describe the variability among the profiles and the corresponding 'peptide loadings' describe the peptides most associated with this variability. Both can be simultaneously visualised in a 'biplot' that will reveal clustering among profile scores and the peptides most over- or underexpressed for these profiles, from the loading directions. The profiles were normalized to unit length before analysis, to reduce the large variation of several profiles that would otherwise dominate the model and the peptide reactivities were then autoscaled, to equalize the potential influence each peptide has on the model. PCA was performed using the Statistics Toolbox for MATLAB (v. R2013a, the Mathworks, Natick MA).

Supplementary Tables

Supplementary Table 1. Comparison of additional parameters with ACPA profiles.

							ACPA isotype[a]					
**Group**	**n**	**female (%)[1]**	**median Ritchie score[2]**	**median swollen joint count[3]**	**median ESR (mm/h)[4]**	**median CRP (mg/l)[5]**	**IgA (%)**	**IgM (%)**	**IgG1 (%)**	**IgG2 (%)**	**IgG3 (%)**	**IgG4 (%)**
**C**	15	80	7.0	9.0	30	12.5	100	83	100	100	83	100
**D**	83	64	9.0	8.0	37	19.5	90	82	98	94	75	100
**E**	30	60	6.5	6.0	35	19.0	31	54	100	77	15	92
**F**	198	67	7.0	9.0	32	16.0	41	22	100	70	30	100
**H**	17	77	8.0	9.5	34	17.0	[b]	[b]	[b]	[b]	[b]	[b]

[a]Only measured in anti-CCP2-positive early arthritis patients; [b]group H only contains anti-CCP2-negative early arthritis patients

Group differences were tested with Pearson's chi-squared test or the Kruskal-Wallis test; p-values: [1] p = 0.582; [2] p = 0.422; [3] p = 0.395; [4] p = 0.278; [5] p = 0.797

ACPA, anti-citrullinated protein/peptide antibodies; CRP, C-reactive protein; ESR, erythrocyte sedimentation rate

Supplementary Table 2. Citrullinated antigens used in multiplex ACPA assays.

Citrullinated antigen	Sequence	Reference	Used in present study[g]
Alpha-fibrinogen	[c]	[21,36]	-
Histone 2B	[c]	[21]	-
Keratin	[c]	[36]	-
Vimentin	[c]	[21]	-
Alpha-enolase 5-21[a]	KIHA**X**EIFDS**X**GNPTVE	[19,21,38]	+[a]
Alpha-fibrinogen 31-50	GGGV**X**GPRVVERHQSACKDS	[20]	+
Alpha-fibrinogen 41-60	E**X**HQSACKDSDWPFCSDEDW[d]	[21,36]	-
Alpha-fibrinogen 172-191	DIDIKIXSC**X**GSCS**X**ALA**X**E	[36]	-
Alpha-fibrinogen 211-230	DLLPS**X**DRQHLPLIKMKPVP[d]	[21]	-
Alpha-fibrinogen 556-575	NTKESSSHHPGIAEFPS**X**GK[d]	[21,38]	-
Alpha-fibrinogen 591[b]	[b]	[38]	+
Alpha-fibrinogen 616-636	THSTK**X**GHAKS**X**PV**X**DCDDVL[d]	[20,21,36]	-
Alpha-fibrinogen 621-635	**X**GHAKS**X**PVXGIHTS	[19]	-
Apolipoprotein E 277-296	A**X**LKSWFEPLVEDMQ**X**QWAG[d]	[20,21]	-
Beta-fibrinogen 36-52	NEEGFFSA**X**GHRPLDKK	[19,38]	+
Beta-fibrinogen 60-74	**X**PAPPPISGGGY**X**A**X**	[19]	+[h]
Beta-fibrinogen 62-81	APPPISGGGY**X**ARPAKAAAT	[19,38]	-
Beta-fibrinogen 62-81	APPPISGGGYRA**X**PAKAAAT	[19,38]	-
Beta-fibrinogen 563-583	HHPGIAEFPS**X**GKSSSYSKQF	[19]	-
Beta-fibrinogen 580-600	SKQFTSSTSSYN**X**GDSTFESKS	[19]	-
Biglycan 247-266	EDLL**X**YSKLY**X**LGLGHNQIR[d]	[21]	-
CCP1	SHQCEST**X**GRSRGRSCGRSGS	[19,38]	+
Clusterin 221-240	PKS**X**IV**X**SLMPFSPYEPLNF[e]	[21]	-
Clusterin 334-353	AEXLT**X**KYNELLKSYQWKML	[20]	-
Collagen type II (citC1[III]) 359-369	(GPO)5-GA**X**GLTG**X**PGDA(GPO)2-GKKYG	[19,38]	-
Histone 2A 1-20	MSG**X**GKTGGKA**X**AKAKS**X**SS[d]	[21]	-
Histone 2B 62-81	IMNSFVNDIFE**X**IAGEAS**X**[d]	[21]	-
Profilaggrin 293-310	TIHAHPGS**XX**GGRHGYHH	[20]	-
Profilaggrin 293-310	TIHAHPGS**X**RGG**X**HGYHH	[20]	-
Vimentin peptide 1-20	MST**X**SVSSSSY**XX**MFGGPGT[f]	[19,36,38]	+[h]
Vimentin peptide 58-77	GVYAT**X**SSAV**X**LXSSVPGV[f]	[19-21,36,38]	+[h]

[a]In the study by Hansson and coworkers [19] and in the present study the cyclic version of peptide was used; [b]the sequence of the peptide was not given; [c]total protein sequence; [d]Sokolove and colleagues [21] used cyclic version of peptide in their study; [e]both cyclic as linear peptide were used; [f]Hansson et al.[19] and Brink et al. [38] used vimentin sequences comprising amino acids 2-17 and 60-75, respectively; [g]in the present study peptides with a C-terminal linker (6-aminohexanoic acid) followed by biotin were used; [h]see Table 1 for details of peptides used; X = citrulline residue

Supplementary Figures

Supplementary Figure 1. The 48-spot microarray.

(**A**) The sensorchip consists of a glass slide covered with a 20 nm gold layer, coated with a hydrogel to which streptavidin is linked. Forty eight solutions containing biotinylated peptides are spotted on this surface by continuous flow microspotting. (**B**) In the *i*SPR apparatus the sensorchip (on top of the glass prism) is placed in a flow cell, allowing the automated incubations with patient sera and regeneration of the microarrays. (**C**) *i*SPR image of a microarray at a specific resonance angle. Each spot on the microarray represents an immobilized peptide. Forty-eight regions of interests (ROI) are defined to monitor interactions with each of the peptides. Each ROI has a width, as well as height of 74 pixels. One pixel corresponds to 7.5x7.5 μm.

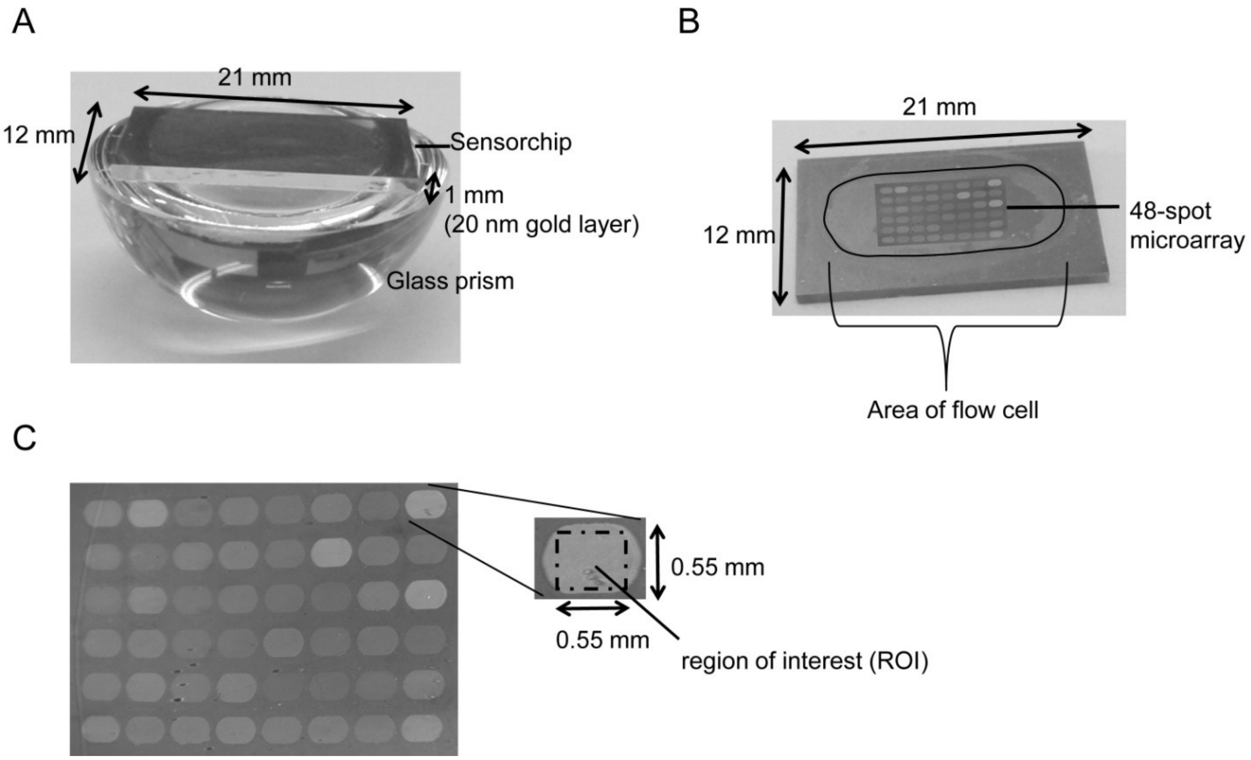

Supplementary Figure 2. The effects of acid-induced microarray regeneration.

(**A**) An example of a measurement, in which the reactivity of 30 analytes (each analysis cycle includes an acid-induced regeneration step) with three different citrullinated peptides was determined. Amongst the different analytes was a mixture of two monoclonal anti-citrullinated protein antibodies (mACPA marked with asterisks). (**B**) The reactivity of this mACPA mixture with three different citrullinated peptides was compared at the beginning and at the end of measurements on eight different microarrays. The ratio of the reactivity at the end (analysis cycle 29) and at the beginning (analysis cycle 1) was determined for each microarray. The graph shows the mean (± SD) for three different peptides (**C**) Reproducibility of microarray analyses (data from 17 microarrays). The reactivity of four different control analytes (as indicated) with a single peptide was monitored. Data is represented as mean ± SD.

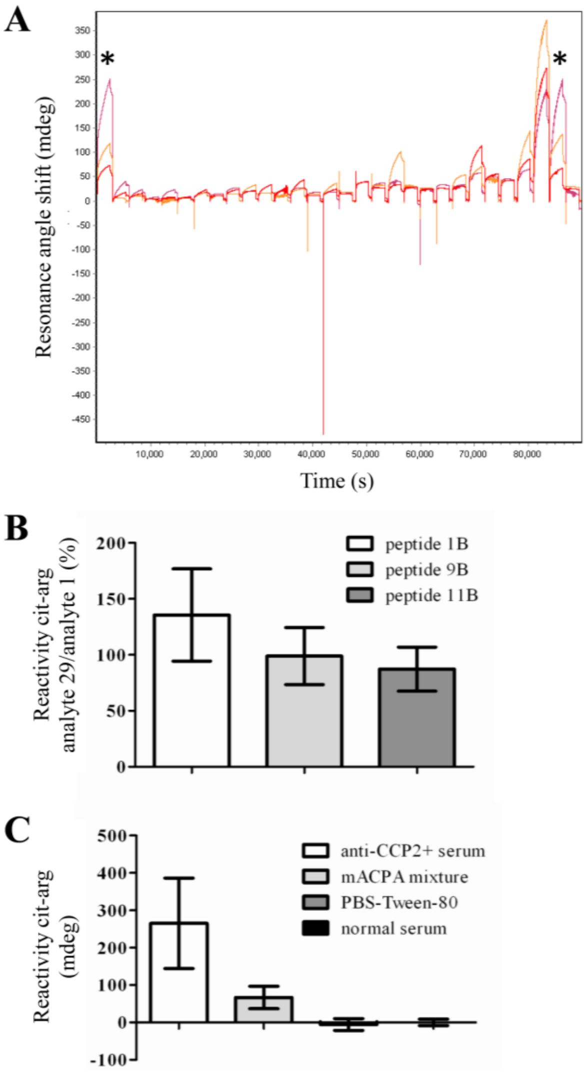

Supplementary Figure 3. Principle component analysis of microarray-*i*SPR data

The data obtained with the first two PCs from a Principal Component Analysis (together describing 47% of the variation in the data) are illustrated in the graph. A-L correspond to the clusters identified with hierarchical clustering (Cluster® 3.0).

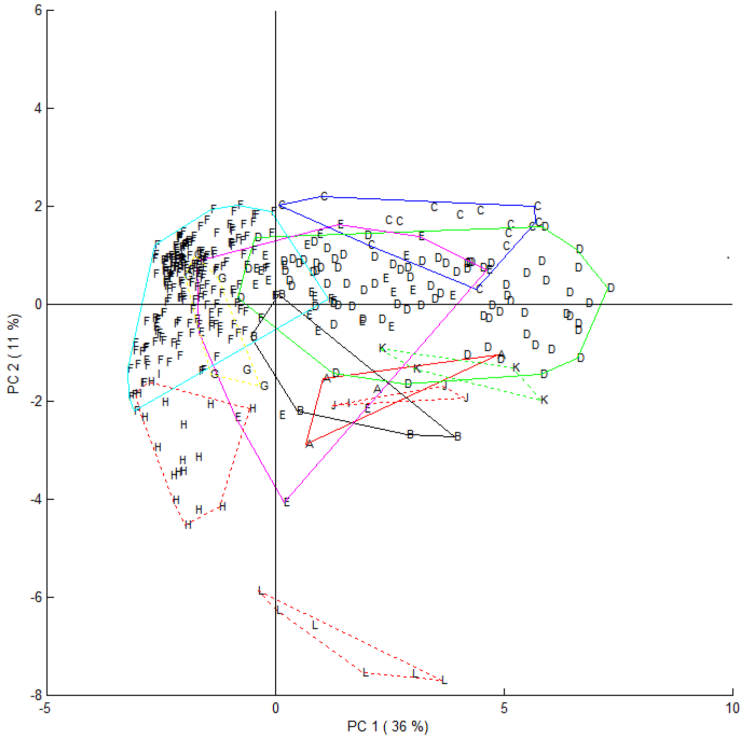

Supplement: Additional file 1 — Supplementary Materials and Methods; Supplementary Tables S1 and S2; Supplementary Figures S1–S3. [file ar4322-S1.pdf]
